# Precision of Disability Estimates for Southeast Asians in the American Community Survey 2008–2010 Microdata

**DOI:** 10.5195/cajgh.2013.40

**Published:** 2013-03-25

**Authors:** Carlos Siordia, Vi Donna Le

**Affiliations:** Preventive Medicine and Community Health, University of Texas Medical Branch

**Keywords:** Southeast Asians, disability, ACS, population estimates, USA

## Abstract

Detailed social data about the United States (US) population were collected as part of the US decennial Census until 2000. Since then, the American Community Survey (ACS) has replaced the long form previously administered in decennial years. The ACS uses a sample rather than the entire US population, and therefore only estimates can be created from the data. This investigation computes disability estimates, standard error, margin of error, and a more comprehensive “range of uncertainty” measure for non-Latino-whites (NLW) and four Southeast Asian groups. Findings reveal that disability estimates for Southeast Asians have a much higher degree of imprecision than for NLW. Within Southeast Asian groups, Vietnamese have the highest level of certainty, followed by the Hmong. Cambodian and Laotian disability estimates contain high levels of uncertainty. Difficulties with self-care and vision contain the highest level of uncertainty relative to ambulatory, cognitive, independent living, and hearing difficulties.

## Introduction

The American Community Survey (ACS), collected by the Census Bureau, is a primary source of population-level disability information in the United States (US). The ACS influences the distribution of millions of dollars, including funding that directly affects people with disabilities. For instance, in the 2008 fiscal year, 184 federal domestic assistance programs used ACS datasets to guide the distribution of $416 billion federal funding.^1^ Data derived from the ACS are used to apportion money for handicapped facilities in mass transit systems and are used in state-level planning for future eligible Medicare and Medicaid recipients (federally funded healthcare insurance).^2^,^3^ The six questions (see Appendix A) in the ACS reflect how disability is conceptualized in the International Classification of Functioning, Disability, and Health.^4^ The questions use a functional perspective to measure “difficulties” with six different areas of daily living so as to assess the disabled and nondisabled population in the US.^4^

Pre-constructed tabulations on the prevalence of disability amongst the different racial-ethnic groups in the US are available.^5^ From the cited source, about 6% of Asians and 13% of NLWs in the US are said to have reported having a disability (i.e., difficulty completing one or more of the six tasks). This data source appropriately warns non-technical readers that caution should be used when comparing population-level measures on specific groups when the statistics are based on small sample sizes and when the margin of error (MOE) is large relative to the given estimate. MOE is a measure of the accuracy in the estimate.

When using sample survey-based estimates to determine population characteristics, the representativeness of the sample—as is the level of precision in the estimate—is of great importance for producing sufficiently meaningful measurements. In this sense, data are said to be unbiased only to the degree that survey-based estimates do not systematically deviate from the ‘true’ population value. The formation of probability samples assumes that all units in the universe have a known, nonzero probability of being selected in the sample—the basic assumption validating the use of inference to generalize from the sample to the target population. In the case of disability estimates for specific populations, the term “estimate” signals that a sophisticated approximation is being made based on a sample and statistical inference.^6^ This means that the estimate contains some uncertainty.

Because accurate information is the primary goal of the US Census Bureau, they produce statistically rigorous data products. The agency provides extensive documentation of their procedures, thereby making imprecision (a measure of sampling error) measurable. Sampling error can be quantitatively estimated with ACS products because replicate weights are provided to public microdata users. Sampling error is the variation between samples drawn from target population where the same random procedure for selection is used. Large variation between samples signals high sampling error and thus that the survey-based estimate may significantly deviate from the true population value. Sampling error operates as a function of sample size, variability in the measure of interest, and the methodology employed in the production of the estimates. Sampling error can be measured using the standard error (SE) of the estimate—sometimes referred to as the MOE. SE measures represent how closely the survey-based estimate approximates the average result of all theoretically possible samples.

This brief report fundamentally argues that before comparisons on disability prevalence between racial-ethnic groups can be made, researchers must evaluate if the “range of uncertainty” (i.e., level of imprecision in the estimate) allows for such comparisons. Accordingly, this exploratory project compares the level of uncertainty in disability estimates between non-Latino-White (standard reference group in the US) and the following Southeast Asian groups: Vietnamese, Cambodian, Hmong, and Laotians. These Southeast Asian groups are selected because they provide a significantly smaller sub-population than the NLWs and may thus have larger variability between the systematically selected samples from the target population (i.e., larger sampling error and thus imprecision).

## Methods

This analysis uses microdata from the ACS 2008–2010 Public Use Microdata Sample (PUMS) file.^7^ ACS PUMS files allow researchers the flexibility to prepare customized variables and tabulations where MOEs and SE around an estimate can also be computed using replicate weights in the microdata. A full discussion on the estimation of MOEs and SEs is given elsewhere.^8^ Confidence intervals on disability estimates, using the standard MOE approach, are provided to show the reader the upper and lower bounds of the estimate—where a 90% confidence level is assured in the measure. To provide a more standardized metric for comparing the precision of disability estimates across the five groups, the following equation is used to calculate the range of uncertainty (RU): [(SE*3) ÷ *x*]*100, where *x* is the estimate. As RU increases, the level of imprecision in the estimate tends to increase.

In addition to these measures, the percent of allocated cases is measured. The ACS PUMS files contain an allocation “flag”—a dichotomous variable of whether or not each response was observed or allocated. An allocation occurs when missing items or inconsistent data are replaced through assignment (fixed using within-person information) or allocation (fixed using outside-person information). For the sake of simplicity, both are referred to as allocations here. Greater details on allocation procedures are available elsewhere.^9^ The percent allocated is determined using the following equation: (weighted allocated count ÷ total weighted population)*100. This measure is given to remind the reader that nonsampling error is not accounted for in the RU measure. Nonsampling error is the practically unmeasurable error that may be created in each stage of the survey process—as when data collection is taking place. In this particular case, nonsampling error is created as a result of nonresponse (when no responses are given) and measurement (when illogical responses are given) errors.

MOEs, SEs, RUs, and percent allocated are calculated for the following disability items: self care, hearing, vision, independent living, ambulatory, and cognitive. For a detailed list of disability related questions, please see Appendix A. The variation of uncertainty in disability estimates in the Southeast Asian groups is examined since the detection of a “Central Asian” group is problematic with the data being used. For example, the dataset does not include any individuals residing in the US and who report being born in Kyrgyzstan, Tajikistan, or Turkmenistan—nor are there any ancestry codes for these places. This may due to the ‘true’ absence of such individuals or to editing protocols that recode their reported place of birth and ancestry into different labels. Please note that previous work has used the US Census Bureau’s definitions with ACS data to create a “South Central Asia” group that includes: Afghanistan; Bangladesh; Bhutan; India; Iran; Kazakhstan; Kyrgyzstan; Maldives; Nepal; Pakistan; Sri Lanka; Tajikistan; Turkmenistan; and Uzbekistan.^11^

Since sample size matters in both weighed counts (using person-weights) and unweighted counts (using actual number of observations). We provide their details. In our analytic sample, NLWs have a weighted count of 148,734,362 and an unweighted count of 4,866,427; Vietnamese have a weighted count of 1,235,633 and an unweighted count of 34,141; Cambodians have a weighted count of 188,749 and an unweighted count of 4,714; Hmong have a weighted count of 129,282 and an unweighted count of 2,779; and Laotians have a weighted count of 164,432 and an unweighted count of 3,945. A rate of inflation (RI) is the average number of persons each individual represents and can be computed by dividing the weighted count by the unweighted number. We note that NLWs have an RI of 30, followed by Vietnamese (36), Cambodians (40), and Laotians (41). Hmong have the highest RI—where, on average, each Hmong survey respondent represent 47 other Hmong.

## Results

Since our specific aim is to evaluate the level of precision in the various disability items across the different groups, we focus our discussion on comparing RUs across groups. NLWs are a standard reference group when investigating the US population, therefore we begin by highlighting that their RUs range from a low 1% to 2% (see Table 1). By comparison, the Vietnamese have RUs ranging from 11% to 14%. From Table 2, we note that Cambodians have RUs ranging from 22% to 44%, Hmong from 30% to 46%, and Laotians from 24% to 93%. As is clear from these numbers, relative to NLWs, all Southeast Asian groups have larger degrees of uncertainty in their disability estimates. Amongst the Southeast Asians, the Vietnamese have the least level of uncertainty in their disability estimates. Cambodians have the largest level of imprecision in the vision item and Hmong in the self care item. The self care and vision disability items amongst Laotians have a comparatively high level of imprecision.

Although it was not the primary focus, we note that disability item allocations for NLWs are at 3%, while allocations ranged from 4% to 5% for Vietnamese, 3% to 5% for Cambodians, 4% to 6% for Hmong, and 3% to 4% for Loatians. These numbers indicate that in general, allocations were more prevalent for all disability items in Southeast Asian groups than NLWs.

## Conclusions

We find that precision levels on the estimates of disability for four Southeast Asian groups are much lower than for NLWs. Since the range of uncertainty qualitatively differs by disability item and Asian group, comparisons of disability prevalence—where ACS data is used—should be avoided or done with great caution. Future work should explore if and how language, amongst Southeast Asian groups, may affect self-reporting of disabilities and how it may impact the rates of allocation with the survey since both introduce imprecision in the estimate.^10^ ACS survey-based disability estimates could potentially affect the quality of services Southeast Asian groups receive, therefore, more research should be undertaken to understand how precision can be improved.

This brief report adds to the literature by poignantly signaling the variability of imprecision on survey-based disability estimates. The level of precision matters and should be accounted for when comparing survey-based estimates between sub-populations. A high level of accuracy in disability estimates is important because they influence US federal funding aimed at aiding the already underserved disabled population.

Unfortunately, investigating small-size sub-populations limits the ability to produce large samples. Under such circumstances, the reduction of imprecision in population estimates may be attempted through improvements in sampling methodologies, survey design, and the administering of the questionnaire. Testing possible solutions is crucial, because most data sources currently used to develop disability estimates make use of sample rather than entire target populations. Because the precision of disability estimates may impact the quality and accuracy of governmental funding, and because the health of the individuals is so closely linked with access to healthcare, more research is needed.

## Figures and Tables

**Table 1:** Weighted number of Non-Latino-White and Vietnamese with disabilities and corresponding estimate precision measures.

	Disable	SE	MOE	LCL	UCL	RU	A
	**Non-Latino-White**						
	
**Ambulatory**	14,439,768	26,505	43,600	14,396,168	14,483,368	1%	3%
**Cognitive**	8,361,648	20,346	33,468	8,328,180	8,395,116	1%	3%
**Hearing**	8,224,412	19,041	31,322	8,193,090	8,255,734	1%	3%
**Independent**	9,932,839	36,643	60,278	9,872,561	9,993,117	1%	3%
**Self-care**	5,585,330	19,082	31,389	5,553,941	5,616,719	1%	3%
**Vision**	4,162,395	22,080	36,321	4,126,074	4,198,716	2%	3%

	**Vietnamese**						
	
**Ambulatory**	58,565	2,508	4,126	54,439	62,691	13%	5%
**Cognitive**	59,223	2,088	3,435	55,788	62,658	11%	5%
**Hearing**	31,431	1,509	2,483	28,948	33,914	14%	4%
**Independent**	58,259	2,248	3,698	54,561	61,957	12%	5%
**Self-care**	29,384	1,489	2,450	26,934	31,834	15%	5%
**Vision**	24,377	1,111	1,827	22,550	26,204	14%	4%

Abbreviations: A, percent allocated= (total weighted population ÷ weighted allocated count)*100; Disable, number of people reporting a “difficulty” with the disability related item; LCL, low limit of 90% confidence interval= (Disability – MOE); MOE, margin of error; RU, range of uncertainty= [(SE*3) ÷ Disability]*100; SE, standard error; UCL, upper limit of 90% confidence interval= (Disability + MOE).

**Table 2:** Weighted number of Cambodian, Hmong, and Laotians with disabilities and corresponding estimate precision measures

	Disable	SE	MOE	LCL	UCL	RU	A
	**Cambodian**						
	
**Ambulatory**	11,795	1,747	2,873	8,922	14,668	44%	5%
**Cognitive**	14,459	1,070	1,761	12,698	16,220	22%	5%
**Hearing**	4,743	530	872	3,871	5,615	34%	3%
**Independent**	12,589	1,266	2,082	10,507	14,671	30%	4%
**Self-care**	4,721	746	1,226	3,495	5,947	47%	5%
**Vision**	5,691	1,103	1,815	3,876	7,506	58%	4%

	**Hmong**						
	
**Ambulatory**	6,518	652	1,072	5,446	7,590	30%	6%
**Cognitive**	7,457	883	1,452	6,005	8,909	36%	6%
**Hearing**	4,267	425	700	3,567	4,967	30%	4%
**Independent**	7,147	808	1,329	5,818	8,476	34%	6%
**Self-care**	3,431	526	866	2,565	4,297	46%	6%
**Vision**	4,229	538	885	3,344	5,114	38%	4%

	**Laotian**						
	
**Ambulatory**	7,814	631	1,037	6,777	8,851	24%	4%
**Cognitive**	8,479	1,248	2,053	6,426	10,532	44%	4%
**Hearing**	4,333	830	1,365	2,968	5,698	57%	3%
**Independent**	9,718	1,244	2,047	7,671	11,765	38%	4%
**Self-care**	4,038	1,044	1,718	2,320	5,756	78%	4%
**Vision**	4,214	1,307	2,150	2,065	6,364	93%	3%

Abbreviations: A, percent allocated= (total weighted population ÷ weighted allocated count)*100; Disable, number of people reporting a “difficulty” with the disability related item; LCL, low limit of 90% confidence interval= (Disability – MOE); MOE, margin of error; RU, range of uncertainty= [(SE*3) ÷ Disability]*100; SE, standard error; UCL, upper limit of 90% confidence interval= (Disability + MOE).

## Appendix A Exact question wording for disability-related items in the American Community Survey

### Self care difficulty

Because of a physical, mental, or emotional condition, does this person have difficulty doing errands alone such as visiting a doctor’s office or shopping?

### Hearing difficulty

Is this person deaf or does he/she have serious difficulty hearing?

### Vision difficulty

Is this person blind or does he/she have serious difficulty seeing even when wearing glasses?

### Independent living difficulty

Does this person have difficulty dressing or bathing?

### Ambulatory difficulty

Does this person have serious difficulty walking or climbing stairs?

### Cognitive difficulty

Because of a physical, mental, or emotional condition, does this person have serious difficulty concentrating, remembering, or making decisions?
